# Translational models for vascular cognitive impairment: a review including larger species

**DOI:** 10.1186/s12916-017-0793-9

**Published:** 2017-01-25

**Authors:** Atticus H. Hainsworth, Stuart M. Allan, Johannes Boltze, Catriona Cunningham, Chad Farris, Elizabeth Head, Masafumi Ihara, Jeremy D. Isaacs, Raj N. Kalaria, Saskia A. M. J. Lesnik Oberstein, Mark B. Moss, Björn Nitzsche, Gary A. Rosenberg, Julie W. Rutten, Melita Salkovic-Petrisic, Aron M. Troen

**Affiliations:** 1 Clinical Neurosciences (J-0B) Molecular and Clinical Sciences Research Institute, St George’s University of London, Cranmer Terrace, London, SW17 0RE UK; 2 Department of Neurology, St George’s University Hospitals NHS Foundation Trust, London, UK; 3 Faculty of Biology, Medicine and Health, University of Manchester, Manchester, M13 9PT UK; 4 Department of Translational Medicine and Cell Technology, University of Lübeck, Lübeck, Germany; 5 Neurovascular Research Laboratory, Massachusetts General Hospital and Harvard Medical School, Charlestown, MA USA; 6 Department of Anatomy & Neurobiology, Boston University School of Medicine, Boston, MA USA; 7 Department of Neurology, Boston University School of Medicine, Boston, MA USA; 8 Department of Pharmacology & Nutritional Sciences, Sanders-Brown Center on Aging, University of Kentucky, Lexington, KY USA; 9 Department of Stroke and Cerebrovascular Diseases, National Cerebral and Cardiovascular Center, Osaka, Japan; 10 Institute of Neuroscience, University of Newcastle-upon-Tyne, Newcastle-upon-Tyne, UK; 11 Department of Clinical Genetics, Leiden University Medical Center, Leiden, Netherlands; 12 Fraunhofer Institute for Cell Therapy and Immunology, Leipzig, Germany; 13 Clinic for Nuclear Medicine, University of Leipzig, Leipzig, Germany; 14 Institute for Anatomy, Faculty of Veterinary Medicine, University of Leipzig, Leipzig, Germany; 15 Department of Neurology, Health Sciences Center, University of New Mexico, Albuquerque, NM USA; 16 Department of Pharmacology, Croatian Institute for Brain Research, University of Zagreb School of Medicine, Zagreb, Croatia; 17 Institute of Biochemistry Food and Nutrition Science, Hebrew University of Jerusalem, Rehovot, Israel; 18 Department of Human Genetics, Leiden University Medical Center, Leiden, Netherlands

**Keywords:** Vascular dementia, Vascular cognitive impairment, VCID, Experimental models, In vivo models, Translational models

## Abstract

**Background:**

Disease models are useful for prospective studies of pathology, identification of molecular and cellular mechanisms, pre-clinical testing of interventions, and validation of clinical biomarkers. Here, we review animal models relevant to vascular cognitive impairment (VCI). A synopsis of each model was initially presented by expert practitioners. Synopses were refined by the authors, and subsequently by the scientific committee of a recent conference (International Conference on Vascular Dementia 2015). Only peer-reviewed sources were cited.

**Methods:**

We included models that mimic VCI-related brain lesions (white matter hypoperfusion injury, focal ischaemia, cerebral amyloid angiopathy) or reproduce VCI risk factors (old age, hypertension, hyperhomocysteinemia, high-salt/high-fat diet) or reproduce genetic causes of VCI (CADASIL-causing Notch3 mutations).

**Conclusions:**

We concluded that (1) translational models may reflect a VCI-relevant pathological process, while not fully replicating a human disease spectrum; (2) rodent models of VCI are limited by paucity of white matter; and (3) further translational models, and improved cognitive testing instruments, are required.

## Introduction

Vascular cognitive impairment (VCI) is a spectrum of clinical disease states [1–4] that range from post-stroke mild cognitive impairment or dementia following a large artery stroke, through ‘sporadic’ small vessel disease (SVD), to pure genetic small vessel arteriopathy (CADASIL, CARASIL, *COL4A1/4A2* mutations) [1, 5, 6]. The most common pathology underlying VCI is cerebral SVD, which leads to focal lacunar ischaemic infarcts, diffuse white matter lesions, and small haemorrhages in deep brain areas [3, 4]. These disease states manifest in a spectrum of cognitive impairments. Further complexity arises as most clinical dementia in older persons is likely to be ‘mixed’ as a result of Alzheimer’s disease (AD) combined with vascular pathology [7, 8]. While characterisation of the neuropathological and radiological features of human VCI has improved over the last two decades (see adjoining articles) the molecular changes that underpin these characteristics remain elusive [6]. VCI currently lacks symptomatic treatment (comparable to donepezil for AD) and molecular targets (comparable to tau, amyloid precursor protein (APP) and β-amyloid (Aβ)).

Because VCI arises from a spectrum of diseases, no single model will reproduce all pathological and cognitive features of SVD or VCI [6, 9–12] (Table 1). Furthermore, as with any animal model for dementia, the behavioural-cognitive phenotype of any given model can never fully represent human cognitive deficits. We define a ‘translational’ model as one that impacts on clinical practice [13]. Therefore, in order to be translational an animal model should reproduce at least one of the pathological processes in human VCI [6, 12, 14]. A fully translational model would permit (1) prospective studies of the timescale and the sequence of events during development of the pathological process, (2) identification of novel molecular, cellular and physiological mechanisms, (3) pre-clinical testing of drugs and other interventions, for proof-of-concept studies, (4) pre-clinical testing of safety profile of drugs, optimal dosing and time-scale, and (5) validation of clinical biomarkers and endpoints such as radiological or biochemical signatures. Models representing the initiating factors would allow translation of preventive strategies, whereas models of advanced disease states allow testing of therapeutic interventions. It is appropriate and timely to seek international accord on such models [15]. Following the recent NIH-sponsored Alzheimer’s Disease-Related Dementias 2016 Summit (https://aspe.hhs.gov/alzheimers-disease-related-dementias-adrd-summit-2016-prioritized-research-milestones), the number one recommendation for VCI was to “*Establish new animal models that: (i) reproduce small vessel disease and other key pathogenic processes thought to result in cognitive impairment; (ii) are easily applicable to both VCID and AD research for advances in mixed etiology dementias; (iii) address vascular contributions to dementia via both white matter and grey matter or (iv) include genetic and acquired conditions that are associated with VCID*”.

**Table 1:** Features of VCI, as related to experimental models considered

	MCAo Rats, mice	MCAo Sheep	Chronic hypo-perfusion Rats, mice	Chronic hypo-perfusion Baboons	HHCy Rats, mice	Chronic HT: SHRSP	Chronic HT: monkeys	Aged dogs	CADASIL mice
Cognitive changes: executive function, attention, processing speed, apathy/reward seeking, memory decline	deficits in spatial and recognition memory; passive avoidance.	post-stroke apathy; higher cognitive function NR	Working memory and reference memory deficits	NR	Impaired spatial learning, working memory	Spatial memory impaired	Reduced executive function, attention, short-term memory	Executive function, spatial learning and memory; visuo-spatial function, simple associative learning; open field activity, anxiety, dis-orientation; restlessness	NR
Sub-cortical motor symptoms: Impaired gait, balance, posture	Sensori-motor deficits. Severity depends on lesion size.	Sensori-motor deficits reflecting lesion size and location	motor deficits on rotarod (GCAS mice).	NR	NA	Sensori-motor deficits. Severity depends on lesion type, location, size	NA	NR	Motor deficits in some aged animals
			No motor deficits reported for BCAS						
Risk factors: age, hypertension, DM, obesity	some studies: age, HT, obesity	NR	HT (SHRSP)	NA	HHCy	HT, dietary risk factors (high fat, high salt); hypo-perfusion	HT	Age (obesity?)	Notch3 mutation
					Co-morbidities e.g., mutant APP				
Brain gross pathology: atrophy, large infarcts..	Focal ischaemic lesion; cortical and striatal	Focal ischaemic lesion; atrophy and pseudo-cyst in chronic stage	NA	NA	NA	Ischaemic lesions and He; variable extent, location	NA	Ventricles enlarged; brain atrophy; spontaneous lesions	NR
Brain neuropathology: Lacunes/micro-Hge, micro-bleeds, diffuse WML	Rapid cell death in ischaemic core. Leukocyte infiltration, neuro-inflammatory changes. Delayed damage in remote areas.	acute cell death in core; inflammatory response; lepto-meningeal and vascular re-organisation; delayed neuroinflammatory response in remote areas	Diffuse WML; micro-Hge; Impaired BBB; microglial activation;	Diffuse WML; microglial activation; Impaired BBB	Micro-Hge in some models	BBB changes, neuro-inflammation.	Focal micro-infarcts; No diffuse WML	Aβ plaques, hippocampal neuronal loss, gliosis, micro-Hge	WML - vacuolisation; focal lesions in some aged animals
						Diffuse WML in animals with UCCAo			
Small vessel changes: Arteriolosclerosis, BBB dysfunction, CAA	NA	NR	CAA in some models	NA	CAA, micro-vascular rarefaction; BBB dysfunction in some models	BBB dysfunction (some studies)	Increased tortuosity	CAA. BBB dysfunction (on MRI)	GOM deposits, impaired CVR; BBB dysfunction (some studies)

Clinical and pathological aspects of VCI are summarised in the first column. How selected animal models relate to these is summarised in the succeeding columns

*Abbreviations*: *BBB* blood–brain-barrier, *CVR* cerebrovascular reactivity, *GOM* granular osmiophilic material, *Hge* haemorrhage, *HHCy* hyperhomocysteinemia, *HT* hypertension, *NA* not applicable, *NR* not reported, *SHRSP* stroke-prone spontaneously hypertensive rats, *UCCAo* unilateral common carotid artery occlusion *WML* white matter lesions

Here, we review published models relevant to VCI, including rodents and emphasising larger species. This review is the result of discussions between experts from 12 laboratories across seven countries. Relevant systematic reviews are available [10, 12].

### Overview of experimental species

#### Rodents

We have included models of focal ischaemia (middle cerebral artery occlusion; MCAo) [16–19] as this is a validated, translational model of cerebrovascular injury. Global hypoperfusion models include bilateral carotid artery occlusion (BCAo) in rats [20] and bilateral carotid artery stenosis (BCAS) using wire coils in mice [21, 22]. A refinement of the BCAo protocol employs constrictor cuffs to give a gradual arterial occlusion over approximately 1–2 days [20]. These global models produce ischaemic white matter lesions, likely reflecting the low baseline perfusion of white matter. Other pathologies can also occur, including hippocampal cell death, small haemorrhages and vascular amyloid deposition. Genetic alterations include inbred strains (e.g., SHR, stroke-prone spontaneously hypertensive rats (SHRSP)) [23–26] or transgenic manipulations (e.g., *Notch3* mutant strains) [27–29]. VCI-relevant animals can also result from manipulation of risk factors, such as age, hypertension, diabetes mellitus, hyperhomocysteinemia or a high-salt/high-fat (‘fast food’) diet [14, 25, 26, 30, 31].

#### Larger species

Larger animals have a longer natural life span than rodents. Experimental ruminants (sheep, goats) are predominantly used to simulate acute cerebrovascular pathologies such as ischaemic stroke [32–34] and cerebral haemorrhage [35]. In domestic dogs, hypercaloric or unbalanced diet, lack of physical exercise and dyslipidemia are prevalent [36]. As in humans, hypertension [37] and cerebral arteriosclerosis [38] are often observed in older subjects. Consequently, a canine cognitive dysfunction syndrome, featuring some clinical aspects of VCI, has been described, particularly in breeds living long enough (>9 years) to fully develop a neurological phenotype [39–42]. In cats, less is known about the relation between aging, vascular pathologies and cognitive decline. Aβ and tau pathologies have been described in cats showing clinical signs of cognitive decline [43–45]. Hypertension associated with arteriosclerosis, as well as small, multifocal cerebral haemorrhages, have also been reported for felines [46].

Behavioural paradigms for cognitive assessment in larger species have been reported from specialist centres for sheep, pigs and cattle [41, 47–51]. The most advanced cognitive abilities are seen in primates, for which sophisticated cognitive tools have been developed [52, 53]. Hypercaloric diet can decelerate aging and prevent microvascular pathologies and cognitive decline in primates [54, 55], without changing the lifespan [56]. Nevertheless, physiological aging can take decades in primates, and studies relevant to VCI may be restricted to specialised colonies [57, 58].

Large animal models allow clinical neuroimaging without significant limitations in resolution, acquisition time or data analysis. MRI protocols are now available for dogs [59], cats [60], non-human primates [61–63], pigs [64, 65] and sheep [66]. MRI (T1, T2, FLAIR) is advantageous for analysis of tissue volume and lesions [66], as well as for anatomical evaluation of particular brain areas [67]. Perfusion and diffusion-weighted sequences reveal cerebral blood flow (CBF) dynamics and vascular permeability [68]. Templates, automatic segmentation and labelling routines for larger species are essential for studies aiming at quantitative morphometric analysis of MRI and/or PET images. Automatic labelling and processing routines have been developed for rhesus and cynomolgus monkeys [61, 69, 70], sheep [67], pigs [71, 72], and dogs [73]; this enables efficient, observer-independent analysis of grey and white matter regions.

## Review methods

For each model, expert practitioners used web-based searches and their own expertise to write a section of the review. All synopses were circulated for editing by all authors, and subsequently by the scientific committee of an international conference (International Conference on Vascular Dementia, ICVD2015, Ljubjiana, Slovenia). Only peer-reviewed sources in English were included.

### Ethical statements on animal data

Sheep experiments from which data were derived were approved by the responsible authorities for University of Lübeck and University of Leipzig, Germany (animal protocol numbers TVV33/09, TVV09/11, TVV33/12). Experiments using monkeys were approved by the Institutional Animal Care and Use Committee of Boston University Medical Center. All procedures with dogs were conducted in accordance with University of Kentucky approved animal protocols (2009-0483) and the NIH Policy on Humane Care and Use of Laboratory Animals.

### Expert reviews of specific models

#### Large Vessel Ischaemia – Middle Cerebral Artery Occlusion (MCAo) in Rodents

MCAo induces acute focal ischaemia bordered by a partially ischaemic penumbra [74, 75]. While recovery of sensorimotor function is well-characterised using behavioural tests, there is less literature on cognitive impairment [76]. Spatial learning, assessed by Y- and T-maze tests, is hippocampus-dependent, but as other regions are also required, including prefrontal cortex and basal forebrain, these tests are relevant to the MCAo model [77]. Following MCAo, male rats showed decreased rates of spontaneous alternation compared with sham-operated animals at day 21 post-stroke [78]. At 4 days post-MCAo, male mice spend less time exploring a novel object than sham animals [79]. Fear-motivated tasks such as passive avoidance have also been used to assess cognitive impairment after stroke [80]. While passive avoidance is a simple task, it is stressful so could confound results of other behavioural tests [76].

#### Larger species: sheep with vascular ischaemic lesions

**Fig. 1:**
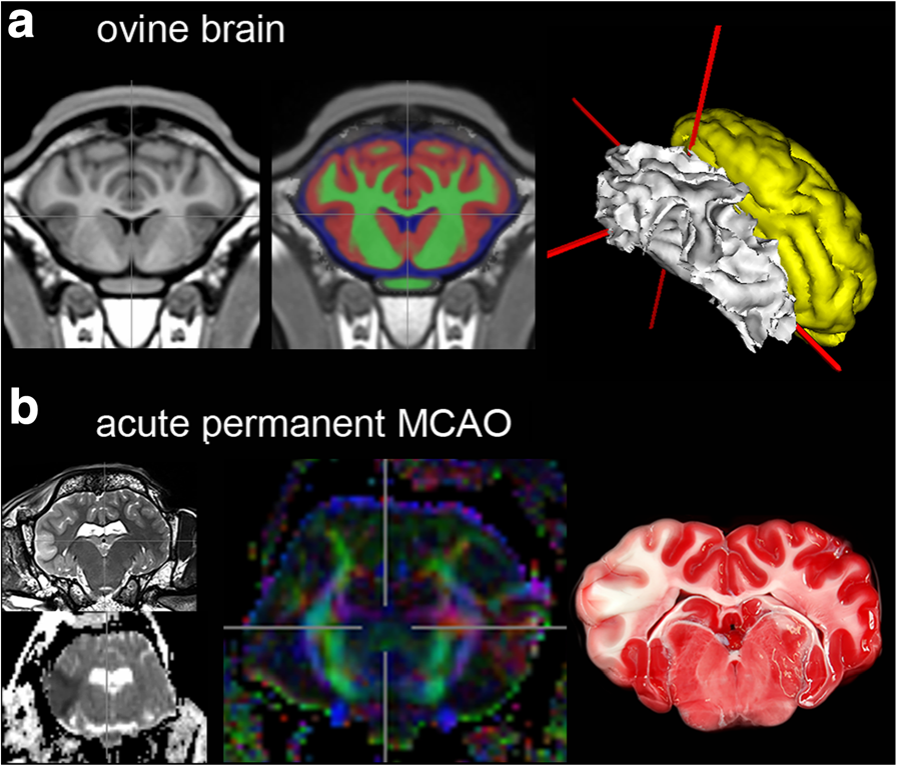
Focal ischaemic lesions in ovine brain. **a** Adult sheep brain in coronal section. T1-weighted population-averaged brain template (*left*), depiction of grey and white matter, as well as cerebrospinal fluid (*middle panel*, overlay on template) and surface reconstruction of white (*white*) and grey matter (*yellow*) in stereotactic space (*right*). *Grey* and *white* matter spaces are derived from a priori tissue probability maps. **b** Focal ischaemic lesion, 6 h after permanent middle cerebral artery occlusion (MCAO). Hyperintense area is seen in the left temporal cortex and medulla in T2-weighted TSE MRI (*left-top*). In this area, a decreased diffusion in apparent diffusion coefficient maps of diffusion weighted imaging (DWI-ADC, *left-bottom*) is visible. Fractional anisotropy map of diffusion tensor imaging (DTI-FA, *middle panel*) reveals a loss of fibre integrity. Following sacrifice and brain removal, the mitochondrial marker TTC labels living cells (*red*). The ischaemic lesion is unlabelled by TTC (*right*)

Permanent [32] and transient [34] MCAo have been performed in sheep, resulting in well controlled and reproducible lesion sizes (Fig. 1). Histopathological investigations revealed both grey and white matter changes, including glial scar formation, microglial activation and replacement of the tissue by new formation of blood vessels and foamy fat cells [33]. Moreover, ovine models have been successfully employed to test experimental therapeutic paradigms in short- [81] and longer-term (up to 7 weeks) approaches [33], during which benefits of single- and multi-mode imaging protocols became evident.

A caveat in this species (and other domestic mammals) is the *rete mirabile epidurale rostrale*, a local arborisation within the carotid artery [82]. This often necessitates a transcranial approach for MCAo. Leaving the trepanation covered only by soft tissue reduces intracranial pressure, which greatly increases long-term survival. In mild and severe global cerebral ischaemia models in sheep, it became evident that the basilar artery can contribute a higher proportion of CBF than in humans [83]. After prior bilateral clamping of both common carotid arteries for 4–30 min, no lesions were found in brains of sheep subjected to the method for less than 10 min. Longer duration produced neuronal changes of several brain regions, similar to those described in other species.

#### Primates and rodents: chronic brain hypoperfusion

With the assumption that reducing CBF is a common feature of VCI [3, 84, 85], the original mouse BCAS model was developed by placing microcoils on the carotid arteries to induce cerebral hypoperfusion [86]. While complete ligation of the carotid arteries (i.e., BCAo) substantially increased mortality, mice can withstand up to 50% BCAS [22, 87]. Monitoring cognitive function using the Y, radial arm, Barnes maze and Morris water maze has provided robust evidence that the BCAS model replicates some features of VCI, in particular the deficit of working memory [10, 86, 87]. In BCAS, global CBF drops rather abruptly. With the same principle as BCAS, ameroid micro-constrictors made of casein (which swells on absorbing water) were placed around the carotid arteries to provide a more gradual stenosis [20]. Ameroid constrictors have also been applied to spontaneously hypertensive rats [20]. Further refinements have allowed the development of mice models that exhibit subcortical infarcts and white matter damage by surgical implantation of an ameroid constrictor to the right common carotid artery and placement of a microcoil to the left common carotid artery to induce approximately 50% arterial stenosis; this is referred to as gradual carotid artery stenosis [88]. There was gradual reduction of CBF over 28 days, and multiple infarct damage in right subcortical regions, including the corpus callosum, internal capsule, hippocampal fimbria, and caudoputamen in 81% of mice [88, 89]. These hypoperfusion models are discussed further elsewhere [12].

A baboon (*Papio anubis*) model evaluated whether partial cerebral ischaemia or oligaemia resulting from reduced blood flow to the brain induces white matter pathology consistent with SVD or AD-like changes. The baboon model is ideal to relate to AD because it exhibits both aβ and tau pathology with ageing and carries *APOE4* associated with AD pathology. Adult, male baboons were subjected to three-vessel occlusion by complete ligation of the internal carotid arteries bilaterally, and occlusion of the left vertebral artery. We have recently reported subcortical and white matter changes in animals to 28 days after three-vessel occlusion [90]. This model is useful to evaluate interventions at various stages and specifically examine the effects of ageing, high-fat diet, hypertension and neuroinflammation. Ameroid constrictors to replicate a gradual reduction in CBF may be a future refinement [84, 85].

#### SHRSP with modified diet or hypoperfusion

Hypertensive rat strains can undergo white matter changes [23–26, 91]. SHRSP typically live for 9–12 months before developing ischaemic and haemorrhagic stroke lesions [12, 92]. When a low-protein, high-salt diet is given to the SHRSP, lesions and death are accelerated [93]. Starting the diet after 6 weeks of life leads to haemorrhagic strokes, but delaying the onset of the diet until the 12th month slows the onset of strokes and allows the damage to the white matter to occur earlier [25]. The white matter damage results from hypoxic hypoperfusion [94]. In a recent study, minocycline, a tetracycline derivative with the ability to inhibit matrix metalloproteinases, reduced white matter damage and reversed the behavioural changes in SHRSP [26]. For a more extensive discussion of SHRSP, see [12, 92].

#### Dietary induction of hyperhomocysteinemia

Elevated circulating homocysteine (hyperhomocysteinemia) is caused by a variety of genetic, physiologic and dietary conditions extensively studied in rodents [95–98]. These cause cognitive impairment in *ApoE* null mice, transgenic mouse models of Alzheimer’s disease, and wildtype mice and rats [31, 99, 100], with surprisingly little neurodegeneration or inflammation. Feeding wildtype C57BL6J mice a diet deficient in three B-vitamins (folate, B12 and B6) for 10 weeks resulted in hyperhomocysteinemia, microvascular rarefaction and impaired performance in the Morris water maze [31, 100]. The same dietary regime in APP transgenic mice worsened cognitive impairment [101], and in combination with excess methionine in dual mutant APP/PS1 mice, the diet induced the redistribution of amyloid from brain parenchyma to the microvasculature along with micro-haemorrhages, as determined by histology and MRI [30, 102]. In Sprague–Dawley rats, folate-deficiency alone was sufficient to induce homocysteinemia and cognitive impairment, and to reduce cerebral blood volume and reactivity measured by absolute, non-invasive, near-infrared spectroscopy [103–105]. For further discussion of hyperhomocysteinemia models, see [12].

Dietary modification can be applied to most species, models and co-morbidities. Caveats are that dietary models typically have higher variability and more subtle effects than genetic or pharmacological models. Outcomes are sensitive to dietary formulation and feeding. This underscores the need for biochemical and metabolic verification of the diet in brain and the periphery. While chronic folate and B12 deficiency in humans causes macrocytic anaemia and myeloneuropathy, these outcomes are almost never observed in rodent models. Associations between microvascular rarefaction and cognitive impairment, in the absence of neurodegenerative changes have been observed in other models, including mice fed a high-fat diet [106], aged rats [107], and irradiated rats [108].

#### Primates with chronic hypertension

**Fig. 2:**
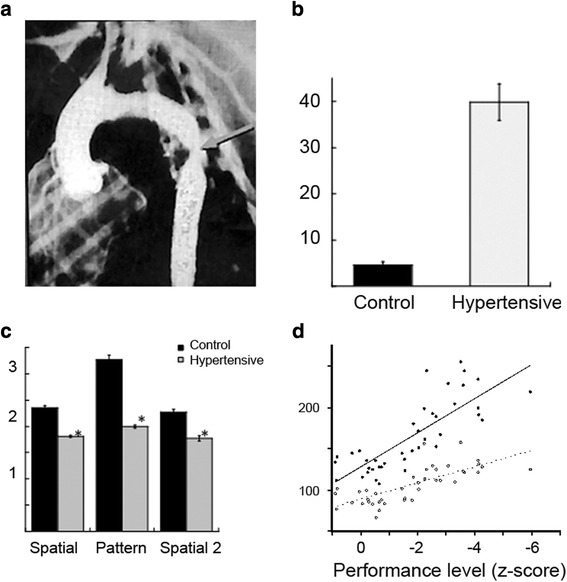
VCI in adult monkeys with surgically-induced chronic hypertension. **a** Arteriogram showing surgical coarctation of the thoracic aorta (*arrow*) in the monkey. **b** Delayed non-matching to sample (DNMS) scores for re-acquisition of the basic task. Y-axis: errors to criterion for control (sham-operated, *black bar*) and hypertensive monkeys (*grey bar*). **c** Delayed recognition span (DRS) test scores. Y-axis: group mean span, for control (*black bars*) and hypertensive monkeys (*grey bars*). **d** Blood pressure correlates with overall cognitive function. Y-axis: blood pressure (mmHg). X-axis: cognitive function index. The level of impairment on this index was significantly and linearly related to both systolic (*black symbols*, *solid line*; *r* = 0.80, *P* < 0.005) and diastolic blood pressure (*open symbols*, *dashed line*; *r* = 0.75, *P* < 0.005). Modified from [52] with permission

The basis of this model is the induction of hypertension by surgical coarctation of thoracic aorta in the rhesus monkey [52, 109–111]. A segment of the thoracic aorta is mobilised and dissected without injuring the mediastinal and intercostal branches. The external diameter of the same segment is measured and then narrowed to a luminal diameter of 2.0–2.5 mm (Fig. 2). A pressure transducer inserted into the femoral artery is advanced through the surgical site. Typically, systolic/diastolic pressure is 170/100 mmHg above the coarctation and 80/50 mmHg (normal for rhesus monkeys) below.

Given the known effects of chronic hypertension on attention, memory and executive function in humans, these domains were assessed in adult primates (5–11 years of age). The tasks consisted of an automated task of simple attention, two tasks of memory function, the delayed non-matching to sample task (DNMS) [112, 113] and the delayed recognition span task [114, 115], and a primate analogue to the Wisconsin Card Sort task, the Conceptual Set-Shifting Task (CSST) [116]. Performance was compared with sham-operated controls that underwent every stage of the surgical procedures up to, but not including, narrowing of the aorta. Animals with coarctation were grouped into borderline (135–150 mmHg) or hypertensive (> 150 mmHg).

On the task of simple attention in which monkeys are required to select the same target stimulus on the touch-screen, there was a positive correlation between response time and systolic and mean blood pressure; hypertensive (but not borderline) animals were significantly impaired relative to the sham-operated group. Hypertensive monkeys were impaired on a task that required orienting to, and then responding by touching, a randomly-presented visual stimulus. Unlike normotensive animals, hypertensive monkeys did not benefit from the presentation of a cue that preceded the target stimulus. The effect did not appear to be related to motivational state as there was no difference in the number of missed trials. These findings suggest a reduction in the speed of processing in the stimulus–response chain.

The findings on memory assessment revealed a significant difference among the groups on the DNMS up to 12 months post-surgery. Hypertensive monkeys re-learned the DNMS task less efficiently than sham-operated controls (Fig. 2). On both the spatial and pattern conditions of the delayed recognition span task, the performance of the hypertensive monkeys was significantly impaired with respect to the control monkeys, suggesting that, in addition to affecting attentional function, hypertension produced an impairment in ‘rule learning’.

The CSST requires the monkey to establish a cognitive set based on a reward contingency, to maintain that set for a period of time, and then shift the set as the reward contingency changes. A subset of hypertensive monkeys was unimpaired on the initial phase of the CSST (a simple three choice discrimination). In contrast, hypertensive monkeys were impaired at abstracting the initial concept of colour on the CSST and were subsequently impaired when shifted to the concept of shape, when shifted back to the concept of colour, and again when shifted back to the concept of shape. The findings from this task suggest that the two groups of monkeys were able to learn a stimulus reinforcement contingency at the same rate and that the impairment seen on the CSST is most likely one of abstraction and cognitive flexibility.

Overall, hypertension significantly influenced higher cognitive function. Blood pressure correlated with a composite z-score (similar to an IQ score), suggesting a direct relationship between blood pressure and cognition (Fig. 2).

Various neuropathologies are seen in this primate model, including tortuous small vessels, hemosiderin-filled macrophages and, most conspicuously, micro-infarcts in both grey and white matter [110, 111]. The micro-infarcts are of irregular shape and relatively uniform size (average maximum diameter ~ 0.5 mm). In the grey matter, these lesions were characterised by a total loss of neurons, and in white matter by marked loss of myelinated fibres.

#### Larger species: aged canine model

**Fig. 3:**
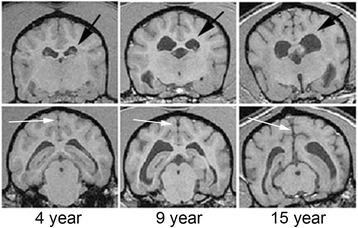
Structural MRI of canine brains. Coronal MRI scans (1.5 Tesla) of 4-, 9-, and 15-year-old dogs, taken from locations at the level of thalamus (*upper row*) and hippocampus (*lower row*). Older animals show marked increase in ventricular volume (*black arrows*) and cortical atrophy, with deep gyri and widened sulci (*white arrows*). Three-dimensional images across the whole brain were acquired using a spoiled gradient recall (SPGR) sequence to obtain detailed anatomic images. Modified from [129] with permission

Aging dogs spontaneously develop cerebrovascular pathology linked to cognitive decline [41, 42], including cortical atrophy and ventricular enlargement (Fig. 3). Cognitive impairment was evident on measures reflecting learning and memory, and a subset of aged animals became severely impaired [41, 42]. A strength of the model is that Aβ, critically involved with plaque accumulation and cerebral amyloid angiopathy (CAA), is very similar in dogs and humans [117–119]. Vascular and perivascular abnormalities and cerebrovascular Aβ pathology are frequently found in aged dogs [40, 120–124]. Dogs may be a suitable model system in which to examine the consequences of CAA on cognition [125]. As in humans, canine CAA is associated with cerebral haemorrhage [40, 121], the occipital cortex being particularly vulnerable [126]. Several environmental manipulations and pharmacological studies that modify lifestyle factors have been successfully implemented in canine models, with some showing significant benefits to cognition [41]. Canines have also been used as a model for ischaemic stroke. Both FLAIR and T2* (sensitive to hemosiderin) imaging show significant white matter hyperintensities [127]. Loss of white matter integrity may be a consequence of CAA; for example, dogs aged from 1 to 20 years exhibited a progressive loss of myelin basic protein, correlated with age and with increasing CAA [128].

The canine brain displays substantial age-associated morphological changes [129–131]. Gadolinium-enhanced MRI revealed reduced blood–brain barrier function with age, as well as reduced cerebrovascular volume [129]. Characterising cognitive function in aging dogs requires many months, and treatment studies may take several years. In comparison to rodent models, they require significant veterinary care as they become older. Radiological outcome measures that reflect in vivo CAA (e.g., SWI scans) have not yet been published.

#### Mouse models for monogenic small vessel disease (CADASIL)

CADASIL (Cerebral Autosomal Dominant Arteriopathy with Subcortical Infarcts and Leukoencephalopathy) is a monogenic archetype for SVD, caused by cysteine-altering missense mutations in *NOTCH3*. CADASIL patients develop progressive white matter lesions from early adulthood, followed by cognitive decline and recurrent subcortical infarctions [132]. Conventional transgenic murine models expressing mutant human *NOTCH3* from a cDNA construct [133–135] recapitulate some aspects of the CADASIL vascular phenotype (vascular Notch3 accumulation and granular osmiophilic material on electron microscopy) [12, 92]. In only one transgenic model, with 4-fold overexpression of mutant Notch3, the mice developed disturbed cerebrovascular reactivity (from 5 months of age), reduced CBF (from 12 months) and white matter damage (from 18 months) [27]. A novel transgenic mouse strain containing genomic human *NOTCH3* has recently been developed [136]; these animals show early-onset vascular Notch3 accumulation (from 6 weeks). A knock-in model, made by introducing a mutation in endogenous *Notch3*, developed a CADASIL clinical phenotype (at 20 months) [137]. Stroke lesions, microbleeds and motor deficits were seen only in a minority of mutant mice (5–12%). Despite the fact that cognition has not yet been characterised in these murine models, they offer a valid pathogenetic representation of human CADASIL and may be an important pre-clinical model in which to test VCI therapies for efficacy.

## Discussion and conclusions

As noted previously [9–11, 14], no experimental model replicates all pathologic and cognitive aspects of human VCI (Table 1). Animal models are useful to reflect a pathological process (e.g., white matter hypoxia, arterial fibrosis, amyloid accumulation) rather than a human disease. Old dogs with canine cognitive dysfunction syndrome and aged primates (> 20 years of age) being possible exceptions, none of the models discussed here results in a ‘demented’ animal. That said, all the animal models considered above reproduce at least one of the pathological processes in human VCI. Because the sequence of events leading from experimental challenge to brain pathology, and thus to VCI, can be characterised in animal models (and interventions imposed), the models may help to identify pathways that lead to VCI. As the pathogenesis of SVD, the most common cause of VCI, remains unknown, a valid model of SVD-dependent VCI remains a challenge. Making these conceptual and biological limitations explicit will expedite the development and appropriate use of translational models for VCI.

There are several general limitations in the extant literature. Most animal studies involve short-term follow-up (typically, less than 4 weeks). Male animals are generally used and females usually avoided due to influences of the reproductive cycle. Few studies have correlated cognitive changes with anatomical changes, as seen by pathology or MRI. Most of the available cognitive paradigms are derived from AD models. Many experimental studies are under-powered (i.e., use a small number of animals) and few are replicated.

We have a number of recommendations for the VCI research community. First, it would be advantageous to increase our knowledge and experience in larger species with more abundant white matter and gyrencephalic brain anatomy. This is especially important given the central role of white matter lesions in human VCI. Second, robust neuropsychological methods for assessing VCI in experimental animals (particularly larger species) would be beneficial. Cognitive impairment (and recovery) are the most complex aspects of human VCI, and will likely differ between animals and humans (for example, experimental species lack spoken language). Thus, aspiring to a precise behavioural replication in an animal may not be possible. Nevertheless, a core toolkit of validated, reproducible, species-appropriate tests of a cognitive phenotype is required. With respect to SVD, simple behavioural indicators analogous to the key cognitive features of the syndrome in humans (impaired processing speed, apathy and executive dysfunction) should be welcome. Third, progress on translational VCI models will be more rapid if high standards of ‘Methodological quality’ [15] outlined in ARRIVE guidelines [138] and in previous translational consensus documents [139, 140] are followed. Specifically, random allocation of animals to experimental groups and blinded assessment of outcomes was quite rare in earlier studies (prior to 2010) [10]. Future experimental studies should adhere to available guidelines on experimental design, regarding a priori statistical power calculation, randomisation, blinding of observers, and confirmation by at least two independent laboratories [15, 138–140]. It appears likely that negative outcomes of animal studies are rarely published. Fourth, as neuroimaging (particularly MRI) has a central role in human VCI, future pre-clinical studies will be enhanced by brain imaging data. Radiological features (diffuse white matter lesions, lacunar infarcts) are the main clinical biomarkers of SVD. Hence, correlative studies relating MRI to brain pathology in animals will continue to be informative.

Experiments using gyrencephalic species may be costly and long in duration to afford sufficient statistical power. A possible solution is a step-wise approach that employs rodents to study fundamental aspects of cerebrovascular disease common to all species, and large animals to study aspects of VCI that require a large gyrencephalic brain. Extending studies across species will clarify molecular, cellular and physiological events that lead from vascular disease to neuronal injury and cognitive dysfunction in humans, and improve the likelihood of achieving new preventive and therapeutic interventions in VCI.

## Abbreviations

BCAo: Bilateral carotid artery occlusion
BCAS: Bilateral carotid artery stenosis
CAA: Cerebral amyloid angiopathy
CBF: Cerebral blood flow
CSST: Conceptual set-shifting task
DNMS: Delayed non-matching to sample task
MCAo: Middle cerebral artery occlusion
SHRSP: Stroke-prone spontaneously hypertensive rats
SVD: Small vessel disease
VCI: Vascular cognitive impairment
